# Predicting the Consistency of Pituitary Macroadenomas: The Utility of Diffusion-Weighted Imaging and Apparent Diffusion Coefficient Measurements for Surgical Planning

**DOI:** 10.3390/diagnostics14050493

**Published:** 2024-02-25

**Authors:** Rania Mostafa A. Hassan, Yassir Edrees Almalki, Mohammad Abd Alkhalik Basha, Sharifa Khalid Alduraibi, Alshehri Hanan Hassan, Mervat Aboualkheir, Ziyad A. Almushayti, Alaa K. Alduraibi, Mona M. Amer, Ahmed M. Abdelkhalik Basha, Mona Mohammed Refaat

**Affiliations:** 1 Department of Diagnostic Radiology, Faculty of Human Medicine, Zagazig University, Zagazig 44519, Egypt; raniahassan@medicine.zu.edu.eg (R.M.A.H.); maatya@zu.edu.eg (M.A.A.B.); refaatmona96@gmail.com (M.M.R.); 2 Division of Radiology, Department of Internal Medicine, Medical College, Najran University, Najran 61441, Saudi Arabia; 3 Department of Radiology, College of Medicine, Qassim University, Buraidah 52571, Saudi Arabia; salduraibi@qu.edu.sa (S.K.A.); ziyadalmushayti@qu.edu.sa (Z.A.A.); al.alderaibi@qu.edu.sa (A.K.A.); 4 Internal Medicine Department, College of Medicine, King Khalid University, Abha 62529, Saudi Arabia; hhsalim@kku.edu.sa; 5 Department of Radiology and Medical Imaging, College of Medicine, Taibah University, Madinah 42353, Saudi Arabia; maboualkheir@taibahu.edu.sa; 6 Department of Neurology, Faculty of Human Medicine, Zagazig University, Zagazig 44519, Egypt; monaamer2345@gmail.com; 7 Faculty of General Medicine, Saint Petersburg State University, Egypt Branch, Cairo 11646, Egypt; st124866@student.spbu.ru

**Keywords:** pituitary macroadenoma, consistency, MRI, DWI, ADC

## Abstract

Understanding the consistency of pituitary macroadenomas is crucial for neurosurgeons planning surgery. This retrospective study aimed to evaluate the utility of diffusion-weighted imaging (DWI) and the apparent diffusion coefficient (ADC) as non-invasive imaging modalities for predicting the consistency of pituitary macroadenomas. This could contribute to appropriate surgical planning and therefore reduce the likelihood of incomplete resections. The study included 45 patients with pathologically confirmed pituitary macroadenomas. Conventional MRI sequences, DWIs, ADC maps, and pre- and post-contrast MRIs were performed. Two neuroradiologists assessed all of the images. Neurosurgeons assessed the consistency of the tumor macroscopically, and histopathologists examined it microscopically. The MRI findings were compared with postoperative data. According to the operative data, macroadenomas were divided into the two following categories based on their consistency: aspirable (n = 27) and non-aspirable tumors (n = 18). A statistically significant difference in DWI findings was found when comparing macroadenomas of different consistencies (*p* < 0.001). Most aspirable macroadenomas (66.7%) were hyperintense according to DWI and hypointense on ADC maps, whereas most non-aspirable macroadenomas (83.3%) were hypointense for DWI and hyperintense on ADC maps. At a cut-off value of 0.63 × 10^−3^ mm^2^/s, the ADC showed a sensitivity of 85.7% and a specificity of 75% for the detection of non-aspirable macroadenomas (AUC, 0.946). The study concluded that DWI should be routinely performed in conjunction with ADC measurements in the preoperative evaluation of pituitary macroadenomas. This approach may aid in surgical planning, ensure that appropriate techniques are utilized, and reduce the risk of incomplete resection.

## 1. Introduction

Pituitary macroadenomas are tumors originating from the anterior lobe of the pituitary gland, and are defined by their substantial size (>10 mm in diameter). They can be classified as non-functioning (exerting mass effects) or functioning (producing excessive hormones) [1,2]. A typical feature is their propensity for suprasellar extension, which can lead to neurological symptoms that necessitate early intervention [3].

The preoperative assessment of macroadenoma consistency is crucial for optimal surgical planning. Although previously thought to require transcranial approaches for hard tumors, the current evidence favors the endoscopic endonasal transsphenoidal route as the primary surgical modality for most pituitary adenomas, even those with a hard consistency [4,5,6]. The preoperative identification of tumor hardness prompts careful planning and meticulous surgical techniques, particularly when operating around delicate structures, such as the cavernous sinus and arteries [7,8,9,10].

Diffusion-weighted imaging (DWI) and apparent diffusion coefficient (ADC) mapping allow for the noninvasive assessment of tumor cellularity and consistency. The ADC correlates inversely with tumor cellularity; highly cellular tumors exhibit a lower ADC due to restricted diffusion [11,12,13,14]. Preliminary evidence suggests that DWI/ADC can allow for the non-invasive assessment of pituitary macroadenoma consistency, which cannot be reliably determined through conventional MRI alone [15,16]. Hard fibrous adenomas (about 10% of cases) have a higher ADC due to their lower cellularity, while soft adenomas exhibit a lower ADC. The preoperative determination of tumor consistency using DWI/ADC is valuable for surgical planning [17,18,19,20,21].

The objective of the current study was to assess the value of DWI and ADC in the preoperative evaluation of pituitary macroadenoma consistency. This, in turn, assists in making informed decisions regarding surgical planning, ultimately reducing the likelihood of incomplete tumor resection and improving patient outcomes.

## 2. Materials and Methods

### 2.1. Ethical Consent

This study underwent a thorough ethical review process, and received approval from the institutional review board of the Faculty of Medicine, Zagazig University, Egypt (approval number, ZU-11409). The research was conducted in strict compliance with the principles outlined in the Declaration of Helsinki. Given the retrospective nature of the study, the requirement for patient consent was waived. The research team ensured that all ethical considerations and standards to protect patient confidentiality and uphold the principles of medical research ethics were met throughout the study.

### 2.2. Patient Population

This study was conducted between October 2022 and October 2023, and involved a thorough search of our institution’s hospital databases. The search specifically targeted patients referred to the MRI unit by the neurology and neurosurgery departments for suspected pituitary tumors. The research team carefully reviewed the patients’ electronic medical records and case notes to collect comprehensive data for the analysis. The following key aspects of patient information and medical history were examined: demographic data such as age, gender, and other relevant demographic information. Information regarding the clinical investigations performed on patients was documented. The surgical findings of procedures related to pituitary macroadenoma were recorded. Histopathological data, including the confirmation of pituitary macroadenoma, were recorded. Patients with a confirmed pathological diagnosis of pituitary macroadenoma were included in the study. Patients with previous resection and residual lesions (n = 3), microadenomas (tumors with a diameter of less than 10 mm) (n = 4), and those without available histopathological data (n = 1) were excluded from the study. Based on these strict criteria, a total of 45 patients were included in this study.

### 2.3. Imaging Protocol

#### 2.3.1. Patient Preparation

Each patient was instructed to remove all metallic objects from their body. Patients were screened for any medical conditions that might contraindicate the MRI, such as artificial heart valves, metallic stents, cardiac pacemakers, or non-titanium joint prostheses. The procedure, including the duration of the examination, required positioning, and the importance of remaining motionless was thoroughly explained to each patient.

#### 2.3.2. MRI Sequences

The examinations were conducted using a 1.5-Tesla MRI scanner (General Electric Signa Excite, USA and Philips Medical System-Achiva-class II, USA) with a standard head coil. The protocol consisted of the following sequences:

##### Conventional MRI

- Pre-contrast axial, sagittal, and coronal T1-weighted images (T1WI) were obtained with the following parameters: TR, 400–550 ms; TE, 15 ms; field of view (FOV), 250 mm; matrix, 256 × 256; slice thickness, 3 mm; and interslice gap, 1 mm.
- Axial and coronal T2-weighted turbo spin-echo (T2 TSE) images were obtained using the following parameters: TR, 3500–4800 ms; TE, 110 ms; FOV, 250 mm; matrix, 256 × 256; slice thickness, 3 mm; and interslice gap, 1 mm.
- Post-contrast axial, sagittal, and coronal T1WI after the injection of 0.1 mm/kg IV gadopentetate dimeglumine.

##### DWI and ADC Mapping

Prior to contrast administration, breath-hold DWI was performed in the axial and coronal planes, using single-shot spin-echo planar sequences with a parallel imaging technique (TR/TE, 2000/33–55; matrix size, 128 × 128; slice thickness, 5 mm; interslice gap, 1 mm; FOV, 38 cm; b-values, 0 and 1000 s/mm^2^; and scan time, 2 min 47 s). ADC maps were generated automatically, and ADC values were measured using circumferential ROIs (8–50 mm^2^) in the central and solid-appearing portions of the lesions.

### 2.4. Image Analysis

We hypothesized that the imaging features of pituitary macroadenomas, as observed by DWI and ADC maps, might correlate with the tumor consistency. Two experienced neuroradiologists, M.M.R. and R.M.A.H., with 15 and 11 years of experience, respectively, who were blinded to both the operative data and histopathological reports, reviewed all images in consensus. To prepare for this evaluation, both neuroradiologists attended a training session that included two sample cases. Each case was selected to represent one of the different consistencies of adenomas. For each macroadenoma, the radiologists assessed the size, signal intensity, and ADC value of all image sequences.

### 2.5. Reference Standard

All patients in this study underwent surgery to remove the pituitary macroadenomas. All operations were performed by experienced neurosurgeons at our institution. The majority of adenomas (n = 39) were removed using the endoscopic transphaeroidal approach. This technique primarily involves the removal of the majority of the tumor in the midline via suction and curettage. Six adenomas required a more extensive approach, and were thus treated with additional transcranial surgeries. During the operation, neurosurgeons assessed and reported the consistency of each macroadenoma. The macroadenomas were categorized into one of the two following groups based on their consistency: aspirable tumors, which included tumors with soft and intermediate consistencies that were easily aspirated, and non-aspirable tumors, consisting of hard tumors that were difficult to aspirate or required en bloc removal. After surgery, each tumor specimen underwent a detailed histological examination, which was routinely performed. The slides were stained with hematoxylin and eosin, and immunohistochemical analysis was performed using pituitary hormone-specific antibodies. All surgical data and histopathological reports were retrospectively reviewed and compared with preoperative MRI findings to determine whether preoperative MRI could predict surgical outcomes in terms of tumor consistency.

### 2.6. Statistical Analysis

The collected data were digitized and analyzed by computer using IBM SPSS version 23.0 for Windows (SPSS Inc., Chicago, IL, USA). Categorical data are presented as numbers and percentages, and were compared using chi-square or Fisher’s exact tests. For continuous variables, t-tests for independent samples were used for comparison. Multiple regression analysis was performed to assess the relationship between macroadenoma consistency and DWI, ADC, T1WI, T2WI, and tumor morphology. ROC curves were used to determine the optimal cut-off value and accuracy of ADC maps to determine the consistency and resectability of macroadenomas. The threshold for statistical significance was set at *p* < 0.05.

## 3. Results

### 3.1. Patient Population

The final cohort of our analysis comprised 45 patients. The age of the patients ranged from 4 to 55 years, with a mean age of 32.1 ± 16.1 years. The age distribution was as follows: under 20 years (9 patients, 20%), between 20 and 40 years (25 patients, 55.6%), and between 40 and 60 years (11 patients, 24.4%). There were 16 male (35.6%) and 29 female (64.4%) patients. Clinically, a headache was the most common symptom (44.4%), followed by visual disturbances (31.1%), galactorrhea (20%), and acromegaly/short stature (2.2%) (Table 1). After reviewing the operative data, macroadenomas were categorized into one of the two following groups based on their consistency: aspirable tumors (n = 27), and non-aspirable tumors (n = 18).

### 3.2. Conventional MRI Findings

Table 2 presents the conventional MRI findings. Most macroadenomas were well defined (66.7%) and hyperintense on T2WI (73.3%).

### 3.3. DWI and ADC Findings in Relation to Macroadenoma Consistency

**Figure 1:**
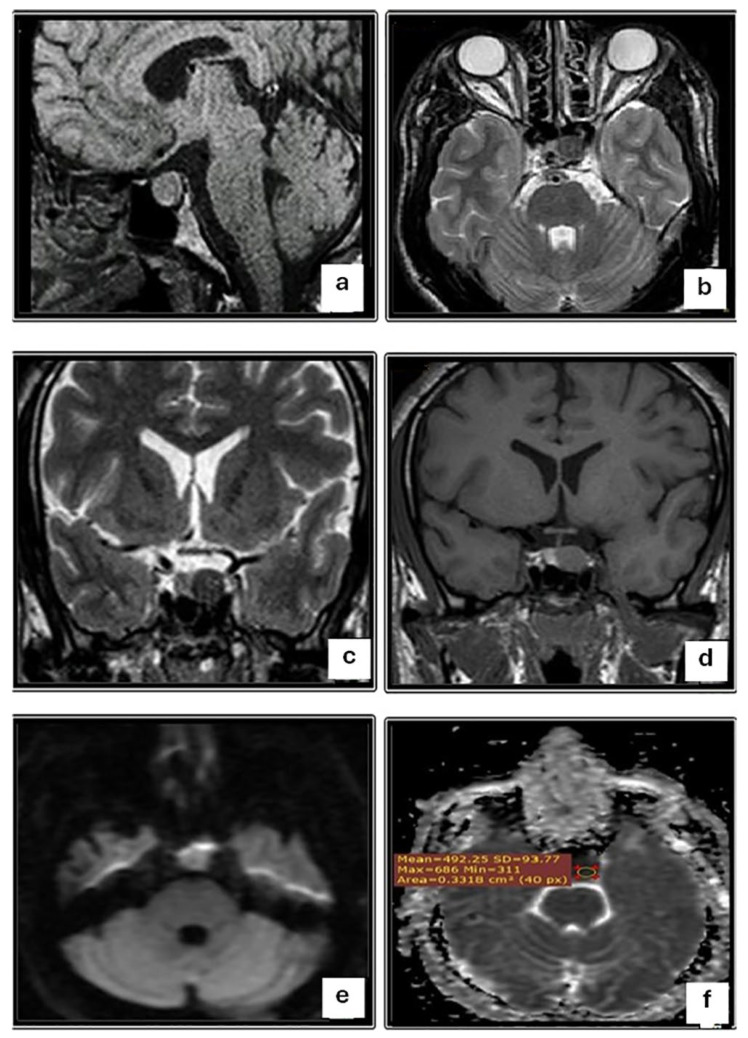
A 50-year-old male patient complains of a persistent headache, progressive enlargement of his hands and feet, and changes in facial features. (**a**) Sagittal T1-weighted image shows a small, well-defined, expanding, hyperintense sellar lesion. No supra or parasellar extensions were observed. (**b**) Axial and (**c**) Coronal T2-weighted images show a hypointense signal of the sellar lesion. (**d**) Coronal contrast-enhanced T1-weighted image shows the homogeneous enhancement of the lesion. (**e**) Axial DWI sequence (b = 1000) shows a hyperintense signal of the sellar lesion. (**f**) Axial ADC map shows a hypointense signal of the sellar lesion with a mean ADC value of 0.49 × 10^−3^ mm^2^/s. The MRI findings are consistent with pituitary macroadenoma. Surgery and histopathology were performed, and a macroadenoma with a soft consistency was confirmed.

**Figure 2:**
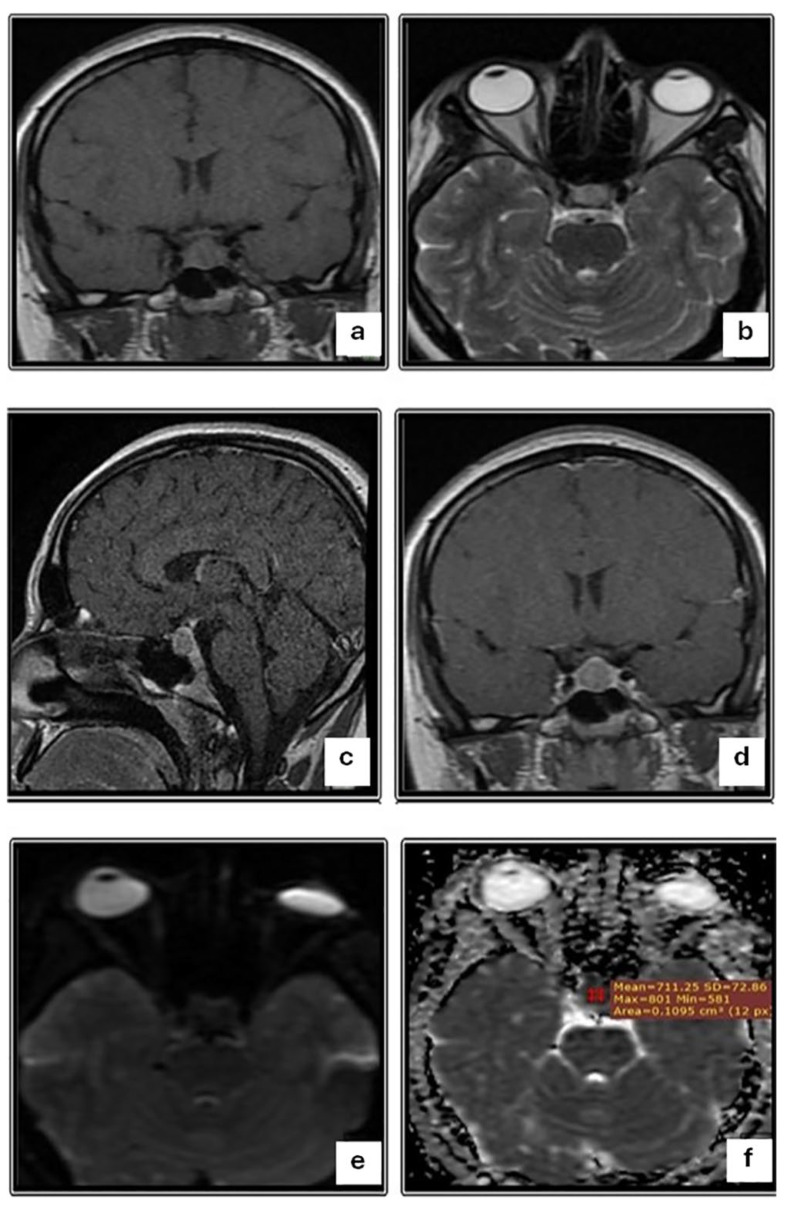
A 35-year-old female patient complaining of a persistent headache. (**a**) Coronal T1-weighted image shows a small, well-defined, expanding, isointense sellar lesion. (**b**) Axial T2-weighted image shows a hyperintense signal of the sellar lesion. (**c**) Sagittal and (**d**) Coronal contrast-enhanced T1-weighted images show the homogeneous enhancement of the sellar lesion. (**e**) Axial DWI sequence (b = 1000) shows an isointense signal of the sellar lesion. (**f**) Axial ADC map shows an isointense signal of the sellar lesion with a mean ADC value of 0.7 × 10^−3^ mm^2^/s. MRI findings are consistent with pituitary macroadenoma. Surgery and histopathology were performed, and a macroadenoma with an intermediate consistency was confirmed.

Table 3 shows the DWI and ADC findings in relation to the consistency of macroadenomas. There was a statistically significant difference in DWI findings when comparing macroadenomas of different consistencies (*p* < 0.001). Most aspirable macroadenomas (66.7%) were hyperintense according to DWI (b100) (Figure 1), whereas most non-aspirable macroadenomas (83.3%) were hypointense. On ADC maps, 66.7% of aspirable macroadenomas were hypointense, while 83.3% of non-aspirable macroadenomas were hyperintense (Figure 2). Aspirable macroadenomas had significantly higher mean ADC value (0.95 × 10^−3^ mm^2^/s) than non-aspirable (0.54 × 10^−3^ mm^2^/s) (*p* = 0.008).

Multiple regression analysis revealed that the ADC map was a statistically significant predictor of macroadenoma consistency (*p* < 0.0001, r = 0.566, r^2^ = 0.782), whereas DWI, T1W1, T2W1, and tumor morphology were not statistically significant predictors of tumor consistency.

### 3.4. ROC Curve

**Figure 3:**
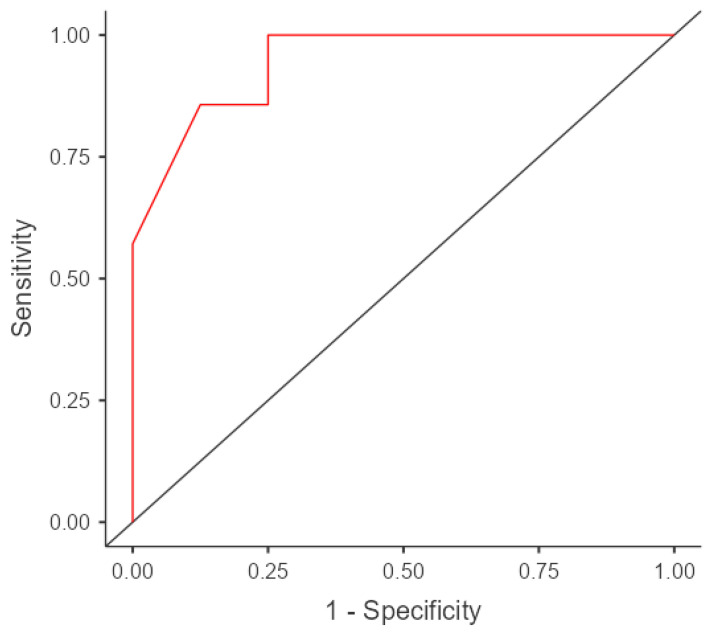
ROC analysis for differentiating aspirable from non-aspirable macroadenoma.

ROC curve analysis differentiated between aspirable and non-aspirable macroadenomas (Figure 3). At a cut-off value of 0.63 × 10^−3^ mm^2^/s, the ADC showed a sensitivity of 85.7%, a specificity of 75%, and an overall accuracy of 80% for the identification of non-aspirable macroadenomas (AUC, 0.946).

## 4. Discussion

Understanding the correlation between tumor consistency and surgical interventions is crucial for optimizing patient care and achieving successful resection. As highlighted by prior studies, and as corroborated by our findings, pituitary macroadenomas with a harder consistency pose greater technical challenges during resection, even with the preferred endoscopic endonasal approach at experienced centers. The preoperative identification of hard tumors enables appropriate preoperative planning and ensures meticulous surgical techniques, particularly when operating around neurovascular structures [22,23,24]. Several studies have highlighted the importance of preoperative imaging, particularly DWI and ADC measurements, in assessing the consistency of pituitary macroadenomas [25,26,27,28]. In line with previous studies, our study demonstrated a significant association between DWI signal characteristics, ADC values, and surgically assessed tumor hardness.

Our analysis of conventional MRI revealed that a significant proportion of macroadenoma lesions exhibited well-defined morphology (66.7%) and hyperintense signals (73.3%) according to T2WI. These findings are consistent with those of Gupta et al. [29], who reported that 63.6% of lesions during T2WI showed heterogeneous hyperintensities. However, our results appear to contradict the observations of Mohamed and Abouhashem [15], who noted a predominance of hypointensity during T2WI in their cases. These discrepancies highlight the variability in MRI characteristics across different studies, and underscore the importance of comprehensive evaluation in clinical practice. Boxerman et al. [20] identified several parameters that can influence T2-weighted signal intensity in macroadenomas. These parameters include tumor cellularity, the presence of free water in the extracellular space, the nuclear/cytoplasmic ratio, the fibrous content, and microcystic components within the tumor.

In our study, we observed that 66.7% of patients with aspirable macroadenomas exhibited a hypointense signal intensity on the ADC map and a hyperintense signal during DWI. This pattern is indicative of restricted diffusion, which is typically associated with a higher cellularity. In contrast, 83.3% of patients with non-aspirable macroadenomas displayed a hyperintense signal on the ADC map and a hypointense signal during DWI, representing unrestricted diffusion, which is often associated with lower tumoral cellularity. The measured ADC values in our study ranged from 0.4 × 10^−3^ to 1.1 × 10^−3^ mm^2^/s, with a mean of 0.72 ± 0.25 × 10^−3^ mm^2^/s. Specifically, patients in the aspirable group (n = 27) had a mean ADC value of 0.54 ± 0.19 × 10^−3^ mm^2^/s, while patients in the non-aspirable group (n = 18) had a mean ADC value of 0.95 ± 0.08 × 10^−3^ mm^2^/s. These findings are consistent with those reported by Mohamed and Abouhashem [15]. Furthermore, our study supports the association between ADC values and tumor consistency, as demonstrated by Thomas et al. [21]. They found that the mean ADC value was 0.75 × 10^−3^ mm^2^/s in the soft group, 0.963 × 10^−3^ mm^2^/s in the intermediate group, and 1.31 × 10^−3^ mm^2^/s in the hard group. However, it is worth noting that their ADC values were higher on average than those observed in our study.

In our study, we identified an ADC cut-off value of 0.63 × 10^−3^ mm^2^/s that effectively distinguished aspirable tumors from non-aspirable tumors. This approach yielded a sensitivity of 85.7%, a specificity of 75%, and an overall accuracy of 80%. Our cut-off value was based on the significant association we observed between DWI, ADC parameters, and macroadenoma consistency. Notably, Mohamed and Abouhashem [15] proposed a slightly lower ADC cut-off value of 0.6 × 10^−3^ mm^2^/s, which exhibited a remarkable sensitivity of 100% and a specificity of 88% in distinguishing between hard and soft/intermediate macroadenomas. In another study by Pierallini et al. [26], 22 patients with pituitary macroadenoma were investigated, and it was found that adenomas with a hard consistency had significantly higher ADC values. They suggested an ADC cut-off value of 1 mm^2^/s to differentiate between aspirable and non-aspirable tumors. However, Suzuki et al. [30], who focused exclusively on cases with soft and intermediate consistencies, reported no hard macroadenoma cases. They observed only marginal differences in the mean ADC values between intermediate and soft tumors, with no significant differences among the groups. Suzuki et al. [30] also found that only two tumors, one intermediate and one soft, exhibited ADC values larger than 1.0 × 10^−3^ mm^2^/s. Discrepancies in previous studies, such as that of Suzuki et al. [30], can be attributed to factors such as the use of modified DWI techniques and limited sample sizes. The choice of imaging techniques and the characteristics of the study population can greatly influence the results. Modified DWI techniques can introduce variations in the data acquisition and interpretation. Additionally, small sample sizes may have led to limited statistical power and potential bias. These variations in findings emphasize the complexity of characterizing pituitary macroadenomas and underscore the importance of considering multiple studies to develop a comprehensive understanding of the tumor characteristics.

The findings of the multiple regression analysis in the current study shed light on the predictive value of various imaging parameters in determining macroadenoma consistency. Notably, the ADC map demonstrated a strong and statistically significant association with macroadenoma consistency (r = 0.566 (*p* < 0.0001). On the other hand, the other imaging parameters, including DWI, T1W1, T2W1, and tumor morphology, did not exhibit statistically significant associations with tumor consistency. These results align with previous investigations that highlighted the utility of ADC in preoperatively predicting pituitary adenoma texture [26,28,31,32]. The high statistical significance of the ADC map in predicting macroadenoma consistency suggests its potential as a valuable imaging biomarker for the clinical assessment of these tumors. Further research is warranted to explore other potential predictors and validate the findings of this study.

This study had several limitations that should be considered. First, it was a non-randomized retrospective study, conducted at a single center, which may limit the generalizability of the findings to other populations. Additionally, the measurement of ADC values may be inconsistent, particularly in heterogeneous tumors. Although we took measurements to position the ROIs in the augmented solid areas and calculated the average ADC values for each case, variability may still exist. Another important limitation is the presence of susceptibility artifacts, which are common in sellar lesions, and can affect the accuracy of ADC measurements. To address this issue, future studies should consider utilizing techniques, such as periodically rotated overlapping parallel lines with enhanced reconstruction (PROPELLER) and planar imaging without echo (EPI), which may provide clearer imaging and improve assessments. Furthermore, this study lacked longitudinal follow-up and did not include comparative analysis with other imaging techniques, such as magnetic resonance spectroscopy (MRS) and functional MRI (fMRI). As this was a retrospective study, we were unable to perform a prospective follow-up or incorporate additional imaging modalities. Therefore, future research should explore the predictive value of other imaging modalities in the evaluation of pituitary macroadenomas.

## 5. Conclusions

Our study underscores the significance of incorporating DWI and ADC maps in the preoperative assessment of macroadenomas. This integrated approach is crucial in determining the most appropriate surgical strategy. We propose an ADC cut-off value of 0.63 × 10^−3^ mm²/s, which enables accurate differentiation between aspirable and non-aspirable macroadenomas. The use of this cut-off value can assist surgeons in planning and performing the optimal surgical procedure, and potentially improve patient outcomes by allowing a tailored approach based on the consistency of the tumor.

While this study provides valuable insights, its limitations highlight the need for further research with larger prospective cohorts, standardized imaging techniques, and longitudinal follow-ups. Such studies could provide more robust and comprehensive evaluations of pituitary macroadenomas, including the assessment of other imaging modalities, for improved diagnostic accuracy.

## Figures and Tables

**Table 1:** Demographic and clinical data of patients.

Variable	Value
Age, years	
0–<20	9 (20)
20–<40	25 (55.6)
40–<60	11 (24.4)
Sex	
Male	16 (35.6)
Female	29 (64.4)
Clinical presentations	
Headache	20 (44.4)
Visual disturbance	14 (31.1)
Galactorrhea	9 (20.0)
Recurrent vomiting	7 (15.6)
Amenorrhea	3 (6.7)
Diabetes insipidus	3 (6.7)
Short stature	1 (2.2)
Acromegaly	1 (2.2)

Data represent the number of patients, with percentages in parentheses. N.B. A single patient may present with more than one presentation.

**Table 2:** Conventional MRI findings.

Finding	Value	*p*-Value
Maximum tumor size, mm, Mean ± SD (range)	25.7 ± 6.9 (18–45)	
Morphology		0.07
Well defined	30 (66.7)	
Ill defined	15 (33.3)	
T1W1		0.01
Iso-intense	18 (40.0)	
Hypo-intense	18 (40.0)	
Hyper-intense	9 (20.0)	
T2W1		0.001
Iso-intense	0 (0)	
Hypo-intense	12 (28.6)	
Hyper-intense	33 (73.3)	

Unless otherwise indicated, data represent the number of patients, with percentages in parentheses. MRI, magnetic resonance imaging; T1WI, T1-weighted image; T2WI, T2-weighted image.

**Table 3:** DWI and ADC findings in relation to consistency of macroadenoma.

	Macroadenoma			*p*-Value
	Total (n = 45)	Aspirable (n = 27)	Non-Aspirable (n = 18)	
DWI (b 100)				<0.001
Iso-intense	9 (20.0)	9 (33.3)	0 (0)	
Hypo-intense	15 (33.3)	0 (0)	15 (83.3)	
Hyper-intense	21 (46.7)	18 (66.7)	3 (16.7)	
ADC map				0.004
Iso-intense	9 (20.0)	9 (33.3)	0 (0)	
Hypo-intense	21 (46.7)	18 (66.7)	3 (16.7)	
Hyper-intense	15 (33.3)	0 (0)	15 (83.3)	
ADC value, Mean ±SD (range)	0.72 ± 0.25 (0.40–1.10)	0.54 ± 0.19 (0.42–0.90)	0.95 ± 0.08 (0.90–1.10)	0.008

Unless otherwise indicated, data represent the number of patients, with percentages in parentheses. MRI, magnetic resonance imaging; DWI, diffusion-weighted imaging; ADC, apparent diffusion coefficient; SD, standard deviation.

## Data Availability

The clinical and imaging datasets used and/or analyzed during the current study are available from the corresponding author upon reasonable request.
